# Bridging the gap between research evidence and its implementation in public health practice: case studies of embedded research model

**DOI:** 10.1186/s12889-024-18727-z

**Published:** 2024-05-13

**Authors:** Abisope Akintola, Dorothy Newbury-Birch, Stephanie Kilinc

**Affiliations:** 1 School of Health and Life Science, Teesside University, Middlesbrough, UK; 2 School of School of Social Sciences, Humanities & Law, Teesside University, Middlesbrough, UK; 3 Manchester Institute of Innovation Research, Alliance Manchester Business School, University of Manchester, Manchester, UK

**Keywords:** Public health, Embedded research, Research-based evidence, Co-production, Research evidence- implementation gap

## Abstract

**Aim:**

To investigate the potential of embedded research in bridging the gap between research evidence and its implementation in public health practice.

**Methods:**

Using a case study methodology, semi-structured interviews were conducted with 4 embedded researchers, 9 public health practitioners, and 4 other stakeholders (2 teachers and 2 students) across four case study sites. Sites and individuals were purposively selected. Sites included two local authorities, one secondary school, and one sports organisation. Thematic data analysis was adopted to analyse the qualitative data.

**Results:**

Four themes were identified: (1) building and maintaining relationships, (2) working with stakeholders, (3) informing practice, and (4) critical reflection.

**Conclusions:**

Embedded researchers build and maintain relationships with practitioners and other stakeholders to produce research. Evidence from the co-produced research informs future practice and research to improve service and delivery rendered to the public. Thus, embedded researchers use their role to bridge the research evidence - implementation gap in public health practice.

## Background

Implementation science is widely recognised as a study of methods to adopt and utilise evidence-based interventions in specific locations or settings to improve the health of the population [1]. However, the gap between research evidence and its implementation in public health practice is still globally recognised [2]. According to scholars, some of the factors associated with the problem of inadequate implementation of research evidence in practice could either originate from the researchers or the practitioners [3–5]. This implies that both researchers and practitioners could be responsible for the creation of the gap between research evidence and its implementation in public health practice.

Evidence suggests that lack of access to research evidence is one of the barriers to the implementation of research evidence in practice [6–8]. One report suggests that increased connectivity between researchers and practitioners would enhance the practitioners’ accessibility to research evidence [9]. The report explained further that creating some forums where practitioners and researchers could interact would not only bring about easy access to relevant research evidence, but also would serve as a means to share learning, and link researchers and practitioners who have a common interest. Similarly, other scholars report that increasing the interaction between researchers and practitioners among other factors could facilitate the use of research-based evidence in practice [10, 11]. To that end, there is a need to increase the opportunities for practitioners and researchers to interact in order to facilitate the utilisation of research evidence in public health practice.

As there are many identified barriers to the use of research evidence in practice, the disparity between the context and the language by which researchers and practitioners operate has also been identified as one of the barriers. The incompatibility in the language spoken by the researchers with respect to the scientific methods and the evidence generated could be ambiguous for practitioners [12]. Therefore, to overcome this challenge, scholars advise that practitioners and researchers should work collaboratively from the onset of the research while putting into consideration each other’s differences [13, 14]. Furthermore, it has been recommended that researchers need to present their research findings and explain the relevance to solving practical problems to the practitioners in a simple language without ambiguity [15]. This suggests a need for an approach that would involve practitioners and researchers undertaking the research agenda together, and also a need for effectively communicating research findings and their relevance in a simple language to the practitioners.

The context in which the researchers operate could also serve as a challenge to the utilisation of research evidence in practice [9]. As such, competing pressures such as teaching commitments and publishing academic papers [16] could pose a challenge to the researchers’ involvement in practical problems that could inform their research questions. Hence, there is a need for an approach for researchers to be more involved in practical problems to facilitate the conduction of research that is relevant and applicable to problem solving. It was noted that not all researchers have the relevant skills to conduct co-produced research [17]. There is a need to create opportunities for researchers who have relevant skills to co-produce research, to conduct research with suitable practitioners.

On the other hand, organisational factors such as time constraints are contributing factors to the gap between research evidence and practice as most practitioners do not have the skills nor the time needed to implement research outcomes in practice [18]. To tackle these challenges, some studies recommend continuous training and commitment to quality health delivery on the part of practitioners. They also recommended advancements in technological decision support systems as instruments to combat barriers between research evidence and practice [19, 20]. There is an argument that achieving these may be difficult as a result of inadequate funds in health services [21]. Hence, there is a need for the adoption of a method that will bring about building the capacity of the practitioners towards conducting research that is achievable based on the available budget.

Furthermore, the disparity of influence and power between academics and practitioners could be responsible for the wide gap between research and practice [22]. This means the relationship between academic researchers and practitioners plays a vital role in the use of research evidence. Therefore, there is a need for a method that would enhance or build mutually beneficial relationships between academic researchers and practitioners to bridge the ‘research evidence-implementation’ gap.

The separation of the development of research evidence from the places it is to be used contributes to the challenges of using research evidence in practice [23]. This implies that the creation of research knowledge where it is to be utilised could bridge the ‘research evidence-implementation’ gap. As such, co-production has been recommended by scholars to bridge the ‘research evidence-implementation’ gap as co-production involves the collaborative working between the researchers and the practitioners [24]. Hence, the adoption of co-production to produce public health knowledge by researchers, practitioners, and other stakeholders in non-clinical settings [13, 25]. This is essential in tackling the challenges of inadequate implementation of research evidence in public health settings.

Being involved in co-production could result in reputational risk for the researcher involved as the researcher could be used by politicians to enhance authenticity to their political stand [26]. Thus, being viewed to approve such a political stand can limit the researcher’s ability to work only with a certain political group – this can also impact the researcher’s personal safety [27]. Also, this can impact negatively on the credibility of the co-production findings as it might be viewed as biased and not a true representation but a narrative to back up a political viewpoint, thus generating “policy-based evidence” [28] rather than “research-based evidence”. On the other hand, policy-makers might be at risk of sharing sensitive information while participating in co-production work [29] such as disclosing political errors.

Also, co-production can be costly as it usually involves the stakeholders travelling to the co-production site. This could be viewed as challenging for those that are involved in the co-production project, as their presence at meetings for the co-production work is seen as crucial. Also, funding and sustainability of co-production can pose a great risk to the adoption of co-production [48]. However, the challenges associated with co-production can be overcome if stakeholders are involved and are carried along at every stage of co-production, from design to implementation [30]. The success of co-production depends on but is not limited to the following: the individuals involved; how clear the aims and objectives of the project are to all those involved, and how duties are allocated [31]. This also suggests a need to critically analyse the role of stakeholders involved in co-production to overcome the challenges associated with co-production, to achieve success.

Embedded research, also known as ‘researcher-in-residence’, is becoming popular as a type of co-production research [3]. Different authors used different terminologies for embedded researchers such as insider researcher [32], knowledge broker [33, 34], or scholar-practitioner [35]. Within an embedded research model, one of the distinguishing features is that the researcher is located in the host organisation as a member of staff to carry out a research agenda with the host organisation’s staff, and at the same time maintaining affiliation with an academic institution [36–39]. In this paper we investigate how an embedded research model can help bridge the gap between research evidence and its implementation in public health practice.

## Methods

We conducted qualitative case studies and drew data from semi-structured interviews with four embedded researchers, nine public health practitioners, and four other stakeholders (two teachers and two students) across four case study sites including two local authorities (Sites one and two), one secondary school (Site three), and one sports organisation (Site four) in the Northeast of England.

One of the advantages of qualitative research is the ability to generate rich in-depth data or knowledge that can serve as a basis for health and social practices being effective and relevant to the contexts they are applied to [40]. We adopted a qualitative multi-site case study to understand the context by providing in-depth description and analysis within sites and as well by comparing data between sites in order to identify the similarities and differences between the sites explored [41]. Thus, this will assist to maximise the applicability of the findings on how an embedded research model can help bridge the gap between research evidence and its implementation in other similar settings.

In site one, the embedded research project aimed to understand and make recommendations regarding population changes, and service needs, including health, education, housing, and social care, in the local communities. In site two, an embedded researcher works at the local authority to provide research support to the local authority’s public health team to secure their targets which include commissioning evidence-based services and interventions, and promotion of healthy lifestyles. Site three conducted an embedded research project to explore the academic and health impact of the recent changes to the General Certificate of Secondary Education (GCSE) system on both staff and students. Site four was established to encourage more people to engage in physical activities to improve their health and well-being. In order to improve the service rendered to the public, an embedded researcher was employed in site four to co-produce research with the sports organisation members of staff. All the embedded researchers across the four case study sites were PhD holders. The amount of time spent in their respective host organisations varied from one hour per fortnight to two and a half days a week to suit the embedded researchers and the host organisations. The embedded researchers’ positions were funded either by the University they are affiliated with, or their host organisation.

Purposive snowball sampling was used in this study. Requests for participants and sites who could volunteer to be part of the study were sent out via relevant professional contacts and networks. The participants and sites that volunteered to take part in this research were asked to assist in the search for participants and/or sites by circulating the study’s details to those who might meet the study’s criteria and would be willing to take part in the study. The inclusion criteria were: (1) being a public health embedded researcher, and (2) being a public health practitioner or stakeholder who is working or has worked with a public health embedded researcher. Potential participants were assessed for eligibility before being interviewed. A total of 17 participants were recruited for the interviews across the four case study sites. The sample size would have been larger than 17 but for the Covid-19 pandemic. Ethical approval was obtained from the Teesside University School of Health and Life Sciences Research Governance and Ethics Committee in November 2019. Data was collected between November 2019 and April 2020.

To facilitate participation, participants were offered alternative modes of interview for their convenience: face-to-face, telephone, and Skype-based interviews. The Covid-19 pandemic occurred during the interview period, but most interviews conducted before COVID-19 were face-to-face. All interviews conducted during the pandemic (March 2020 and onwards) were either Skype or telephone-based, as advised by the Ethics department at Teesside University and as per the requirements of the interviewees’ workplaces. Before each interview, oral and written informed consent was obtained from each participant. Each participant was asked to complete two copies of the consent form, one for their own records and one for the researcher.

Following each interview, a reflective note was taken to identify what went well and what could be done differently in the next interview. Since there were three categories of interview participants – embedded researchers (ERs), public health practitioners (PHPs), and other stakeholders (students (STs) and teachers (TRs)–three sets of interviews were prepared. Although the interview questions were nearly the same for each category of participants, some of the interview questions differed in the way they were structured. Here is an example of how a question was worded differently depending on the participant: (ERs) *Can you cite an example where you have built practitioners and other stakeholders’ confidence to conduct their own research?* (PHPs, TRs, and STs) *Can you cite an example where an embedded researcher has built your confidence to conduct your own research?* A full outline of the interview guide is in Appendix.

A summary of each interview was noted in a research diary for reference. Details noted included where each interview took place, the date of the interview, the length of the interview and how the interviewee responded to questions. Each interview lasted between 40 and 90 min. The interviews were recorded, and data was transcribed. We analysed data using inductive thematic analysis [42] to allow new themes besides the preconceived ones to emerge from the coding of the interviews. Trustworthiness of the analysis was assessed by triangulating between data sources.

## Results

Four themes emerged from the analysis of the interview data on the potential of embedded research in bridging the gap between research evidence and its implementation in public health practice: (1) building and maintaining relationships (2) working with stakeholders, (3) informing practice, and (4) critical reflection.

### Building and maintaining relationships

All participants across the four case study sites, irrespective of their age, years of experience, or education, recounted the significance of this theme to the embedded research projects in their respective sites. They articulated the benefits of the role of the embedded researchers in building and maintaining relationships with the public health practitioners and other stakeholders to facilitate the co-production of research evidence. They all agreed that building and maintaining relationships played a vital role in the utilisation of the co-produced research evidence and in the closing of the gap between research evidence and its implementation. Overall, the strategies adopted by the embedded researchers to achieve this theme were identified as: (1) building internal/external relationships and sharing skills, and 2) maintaining regular contact with practitioners and other stakeholders.

### Building internal/external relationships and sharing skills

Participants agreed that the embedded researchers’ role entails having diverse connections built on good relationships. These relationships assist the embedded researchers in connecting their partners to other relevant organisations such as academic institutions and third sector agencies.

*“I think some of that is around having this kind of good grounding so sort of beginning the role with already having made, a lot of kind of contacts, a lot of sort of good relationships been built. [..] I have a line manager in the council, who was the project manager for the first phase so we’ve got that continuity there [..] I also have an academic supervisor who is also my kind of my line manager from the academic side”****[ERsite1]***.

*“I can say that’s [having connections] actually key because they are straddling both worlds. [..] not somebody who sat in the academic institution who didn’t understand the wider context. I think these roles are really key in bridging the institutions”****[PHP2site1]***.

It was clear that building relationships and connecting the ‘two worlds’ is not only advantageous to both institutions but also assisted the embedded researchers to seek support from both their academic supervisor at the University they were employed and the local authority (LA) they are working with. Therefore, this enables the embedded researchers to be supported fully to carry out their role successfully. It was also recognised that while embedded researchers play their role in building relationships and connecting relevant organisations, the role assisted them to understand the context in which research evidence is to be utilised. Thus, the relevance of research evidence to the host organisation facilitates its use.

This relationship-building was seen as crucial to the success of the role, and it was felt that these relationships could determine the success of any work carried out.

*“[..] I would go as far to say I think it’s the relationship that’s built with the individuals who developed that project was important. [..] are the most important elements of co-production”****[ERsite2]***.

This implies that lack of relationship-building between researchers and public health practitioners can serve as a barrier to embedded research project. Furthermore, it was evident that the relationship built with the stakeholders who were involved in the embedded research was crucial to the projects. For instance, an embedded researcher from site two used her skills to build relationships with the volunteers that participated in the project.

*“She [embedded researcher] has been there longer, excellent relationships with the volunteers, that helped to build and shape this project, so she has a very useful experience in terms of relationship-building”****[PHP6site2]***.

Thus, this assisted in structuring the work which had a positive impact on the project. This two-way relationship with other organisations, including the local universities and research participants, was seen as a benefit of embedded research.

Findings showed that embedded researchers used their contacts and good relationships to facilitate the sharing of skills useful in carrying out embedded research projects and also enable working with other academics at the University.

*“[..] even for me just working as an individual in that organisation, I don’t know everything about the research, but because you are linked with the University, that gives an avenue to ask questions and link up with people with expertise to then support an evaluation”****[ERsite2]***.

These connections and relationships, therefore, enable the sharing of skills useful to co-produce relevant high-quality research evidence useful to host organisations and policy makers.

Within this current study, it was clear that if the embedded researchers were not located or had spent time in the sites, they felt it would be difficult for them to build relationships, and understand the context in which the co-produced research is to be utilised.

*“So, having the researcher embedded within in what we do, the researcher has the understanding of the project, and initially she has been with it from the start to finish, so she understands the journey that’s been on, and she understands why it’s been done, how it’s been done [..] So, I think, so the embedded researcher role in what we do is infallible resource really”***[*PHP1site4*]**.

The ‘embeddedness’ gave the researchers an understanding of the projects they were involved in. As such, the embedded researchers were seen as ‘insiders’ and their ‘embeddedness’ was seen as key to the success of the work.

It is worth noting that the amount of time spent by the embedded researchers in their respective host organisation varied and was negotiated at the sites to suit the embedded researchers and the host organisations.

*“[..] I was familiar with quite a lot of people but obviously kind of being there regularly I have got to know them much better basically. [..] I mean it really varies; I would say probably kind of at least a couple of days in a week”****[ERsite1]***.

*“Being embedded within their team I spend half of the week working within the organisation. It’s been a real pleasure to work alongside them”****[ERsite2]***.

***“****So, we tend to have meetings where I will go in for a few hours at a time. I would probably say, maybe an hour in a fortnight****” [ERsite3]***.

*“[..] I spend two and a half days working within the organisation.* [..] *you want to be seen as part of that team and not somebody who just pops up every now and again”****[ERsite4]***.

However, building relationships and sharing skills was not seen as without its challenges with some tension between roles and expectations.

*“[..] it has become trickier splitting myself now between the organisations as they all have their roles and expectations on how they want things to be done” [****ERsite2]***.

*“The structure can be quite challenging as well, but probably [..] just having that balance in the relationships with the organisation you are working for and the organisation you are evaluating for. And I think yeah you have got to have that one, but that is a challenge of working in large organisation”****[PHP6site2]***.

The embedded researchers from sites one and two found there was some tension in working in both ‘worlds’ as a result of the responsibilities associated with it, such as building relationships, and balancing diverse responsibilities. This is due to their dual affiliation as such, they are expected to manage a large workload, managing both successfully. A practitioner from site two added that the structure of the organisations the embedded researcher works could also be a challenge, therefore, it is important for an embedded researcher to be able to discuss this with both sides in order that they balance the relationships between the host organisation and the academic institution.

Another notable challenge is having to manage diverse expectations including the ability to balance competing interests of the different organisations.

*“There is sort of difference in expectations because I think from the academic point of view, [..] we want publications, we want things that give us an academic output, whereas someone who works in the school is not going to be bothered about that sort of things. They have to see where it positively affects their school, [..] so I think having that difference in agendas on what you want to achieve from this school research can be quite hard to manage. [..] you want different things from this piece of research is quite hard, and make sure that both sides are happy at the end of the day, and I think we did that quite well”****[ERsite3]***.

For instance, an embedded researcher from the school stated that the expectations from the embedded research project did differ. That is, while part of the aim of the academic input was to publish the outcome of the project to improve or boost their academic output, the school aimed for a practical positive impact of the project on the school, such as improvement in students’ engagement in academic activities. Hence, it was essential to balance the competing interests of the school and the academic side of the embedded research project.

### Maintaining regular contact with practitioners and other stakeholders

Based on the participants’ experiences, the embedded researchers built relationships with the practitioners and other stakeholders by maintaining regular contact.

*“I think what we did was to help build that relationship. It was not just a telephone conversation just to discuss. We actually worked side by side so there was time to actually do that embedded research. We spent time in the office, we spent like one or two days a week”****[PHP1site2]***.

*“Yeah, but then we did send them emails and stuff, in between [..] yeah we did have time outside of the face to face sessions and sending stuff to the teachers to encourage them, ‘can you remind the students that we have got to do this week’, we have got to get this done by then, so I would say obviously we had the face to face sessions but then we had email correspondence as well”****[ERsite3]***.

The practitioners from site two reported that the embedded researcher maintained regular contact by face to face, or by telephone. They further explained that they worked side by side with the embedded researcher to build relationships. This implies that if the practitioners and the embedded researcher were not chanced to work together, which assisted in maintaining regular contact, it would have been difficult to build relationships. Thus, this widens the gap between academia and practice. The embedded researchers had similar experiences. For instance, an embedded researcher from site three (school) confirmed that she maintained regular contact to build relationships with the students and the teachers by email and face to face. This shows that it is important to develop project strategies in order to maintain regular contact with the practitioners and other stakeholders to build relationships.

According to the embedded researchers, building mutually beneficial relationships was achieved by maintaining regular contact not only with the stakeholders but also with their academic supervisors which enabled the embedded researchers to have the necessary support to achieve their role.

*“I mean knowing that I do have kind of the support at the University to draw on and also have a kind of a good working relationship with my line manager in the council as well really. I don’t feel that I am lacking in any kind of support, which is a good kind of place to be in yeah. So I have monthly meetings in the University and that’s very much really useful in times of keeping track of some of the other parts of my roles so around kind of trying to ensure that we can get some like academic publications and things like that so yeah”****[ERsite1]***.

Another strategy that was mentioned regarding how the embedded researchers maintained regular contact to build relationships with the practitioners and other stakeholders was ‘attending formal meetings’.

*“Interestingly, the researcher has always been on the co-production committee and she attends the meetings, so she is excellent, much better than me because she has been there longer, [..] that helped to build and shape this project [..]”****[PHP6site2]***.

*“So, I have to go to all their team meetings that’s gonna help you form a lot of relationships. Meetings are where the real connection starts to happen. So, you have to invest that time****” [ERsite4]***.

As well as making use of formal meeting, the embedded researchers adopted ‘informal conversations’ to maintain regular contact to build relationships with the public health practitioners and other stakeholders.

*“For me, I am quite like a chatty person and I think that’s like the characteristics of an embedded researcher. You need somebody who is easy to get on with lots of different people. You need to have that ability to do that. Otherwise, you gonna struggle to form a relationship especially if you aren’t there as often as what you would be if it’s a full-time job” [****ERsite4]***.

A practitioner from the sports organisation added that engaging in informal conversations also helped in building a trustworthy relationship with the embedded researcher.

*“[..] We have that relationship and some other things you can visit, particularly when things get tough, it’s easy enough to fall back on different conversations on sport [..] These conversations increase our relationship and trust, we trust each other”****[PHP1site4]***.

The practitioner further explained that he has a good relationship with the embedded researcher and so they engage in informal conversations at difficult times thereby developing a relationship that is based on trust.

### Working with stakeholders

Results showed that the embedded researchers build and maintain relationships with the practitioners, and with other stakeholders in order to effectively work together to produce research. This, therefore, facilitated the production and the use of the co-produced research evidence at the embedded sites and helped close the gap between research evidence and its implementation as results were shared quickly with all those that were involved. All participants across the four case study sites unanimously agreed that this theme is one of the primary roles of an embedded researcher, and the strategies identified include: (1) co-producing research, and (2) building research capacity.

### Co-producing research

The participants confirmed that they worked together to identify, plan, and conduct research intended to help the host organisations improve their services and meet the needs of the communities with which they work.

*“We liaise with the researcher to develop the initial kind of overview of that population [..] the researcher supports us in developing the initial questions, the questionnaire, and the initial research”****[PHP1site4]***.

*“[..] embedding research into the public health team. [..] then helping us to explore the questionnaires. The embedded researcher helps us with the development of that work including the formulae and evaluation for the intervention. We design and develop and embed and undertake the research together. She is very much a part of the team and a core within the team”****[PHP4site2]***.

*“[..] So, really it’s about giving us the exposure to that sort of research. Well, honestly, I have learnt how to conduct research”****[ST1site3]***.

The participants acknowledged that working together to co-produce research with the embedded researchers encouraged adjustments to and engagement with research-related activities. Furthermore, embedded research was considered a cost-effective research approach.

***“****I have been out in a couple of beneficiary interviews with the researcher. Certainly, I would not normally get involved with going out to see clients, but I have gone out a couple of times with the researcher, so that was interesting”****[PHP5site2]***.

*“[..] the embedded researcher worked alongside the public health practitioners [..] how to shape some of the evaluations, including how to be really clear about the methodology, the approach [..] And how to write protocol [..] So, I think that was the aim of it, it was to ensure that we have much more effective and cost-effective research****” [PHP2site1]***.

One public health practitioner reported that she participated in several research activities with the embedded researcher at site two. She recognised that working with the researcher enabled her to do research work that she would not have ordinarily done. This suggests that not working together with practitioners to co-produce research may potentially prevent practitioners from being meaningfully involved in the research process. In such situations, the gap between the development and implementation of research evidence may actually become wider. One practitioner from site one explained that embedded research was adopted in the LA so that the authority could conduct cost-effective research. This only further indicates that having an embedded researcher on-site working collaboratively with practitioners and stakeholders to conduct cost-effective research can help bridge the research implementation gap.

However, it was noted that the process of co-producing research between the embedded researchers and the public health practitioners and other stakeholders also facilitated shared learning.

*“Despite the fact that we went in obviously thinking of teaching them but the fact that we can learn from them about what was important to them, what was important to young pupils in schools, and how to speak to young pupils because that is schooling in itself. [..] and I think also you learn new skills [..] so I think you get sort of practical experience and learn new skills sort of more practical skills I suppose, not just research skills, so yeah that is why I think I say it’s the most important thing”****[ERsite3]***.

*“[..] and when I have been out with staff members, they will ask questions that I would never have thought of asking, because of their knowledge at work. [..] I have been learning a lot as well from the staff, and that shows the importance of doing it together” [****ERsite2]***.

One embedded researcher from site three (school) reported that although their aim was to teach the students how to conduct research, they were able to learn what was important to the young people among other things from the students. Another embedded researcher from site two shared a similar experience and confirmed that during the co-production work, the public health practitioners used their tacit knowledge of their field to ask relevant questions that had not occurred to her. Since the practitioners are more knowledgeable than the researcher regarding actual on-site practices, they added substantial value to the project. This indicates just how much learning is a two-way process, and demonstrates co-production of knowledge which involves the amalgamation of the practitioners’ tacit knowledge and the researchers’ explicit knowledge.

Researchers were explicitly recognised for their ability to co-produce research with the public health practitioners and other stakeholders. Thus, the co-produced research was jointly owned by those involved in the embedded research projects. As the research was co-produced with the intention to assist the organisations to improve the service they render to the public, thus, the embedded researchers’ role assisted in facilitating the utilisation of research evidence. In addition, given the embedded research projects focused on meeting the needs of the host organisations, there were no instances where there were conflicts related to the research emerged.

### Building research capacity

The embedded researchers explained that they conducted training, and other developmental activities to help develop the practitioners’ and other stakeholders’ research skill-set.

*“I have done a kind of number of training sessions with staff and actually with volunteers that will want to get involved in collecting data [..] so I have run workshops, training workshop, so that means that when I go out there for collection the staff can come and do it with me”****[ERsite4]***.

*“[..] another element of my role is to deliver training to staff around the use of data around the benefits of collecting relevant information, how that information can be used to inform practice in decisions and planning and things like that, we just had a conference couple of weeks ago which was very much about kind of sharing the learning and then sort of getting people involved in the work that we do really, so they are my kind of key targets really” [****ERsite1]***.

Research-based training were offered by the embedded researchers in a variety of forms, such as using workshop training, one to one training and through seminars and conferences. For instance, an embedded researcher from site four (sports organisation) reported that she taught the practitioners to collect data at a training workshop that she organised. She explained that this training assisted the embedded research project because it helped the practitioners to get involved in the data collection phase as they had the skills from the training. Similarly, another embedded researcher from site one reported that getting the practitioners involved in the embedded research work facilitated the sharing of learning, which was one of her main goals while working at the LA. This particular researcher trained the public health practitioners to collect data and taught them how research evidence can inform practical decision making.

The participants agreed that working together with the embedded researchers strengthened their ability to conduct high-quality research capable of benefiting their respective organisations.

***“****It also allowed us to utilise and build the capacity of public health practitioners who would often not undertake any research for some time”****[PHP2site1]***.

*“So, it’s more like continuous professional development [..] So, the research skills are learnt such that at the end of the day, next time the research could be conducted independently, even if we didn’t have somebody coming from the outside. That’s the whole approach [..] is for developing public health practitioners to the extent that research can be conducted in a rigorous manner”****[PHP1site1]***.

*“I think probably when I attended two beneficiary interviews with her and just seeing how to speak to people when you are asking them questions so there is a way to ask the questions so that they understand, probably by listening to the researcher at that point I sort of learnt how” [****PHP5site2]***.

As the above suggests, the embedded researchers encouraged some practitioners who would ordinarily not participate in research to engage in research activities. This implies that working together with researchers may be a significant facilitator to building practitioners’ research capacity and closing the research implementation gap. The absence of an embedded researcher may even serve to widen the gap. Indeed, the public health practitioners observed that working with embedded researchers could eventually build their research capacity to independently conduct high-quality research in the future.

Overall, it was clear that the participants were aware of the importance of working together with embedded researchers, and the researchers were acknowledged for their ability to assist greatly with research-related training and support to build their research capacity. It would have been difficult for these organisations to generate high-quality on-site research if the embedded researchers had not been present. Consequently, the embedded researchers helped work to close the research evidence implementation gap.

### Informing practice

The embedded researchers built and maintained relationships with the practitioners and other stakeholders to work together with them to co-produce research. The participants from the four case study sites reflected upon how the embedded researchers informed the sites of relevant research-based evidence, which helped in the development of future practice and research. By doing so, the embedded researchers bridged the gap between the discovery and implementation of research-based evidence. The results showed that all participants across all the four case study sites, irrespective of age, years of experience, and education, agreed that the role of the embedded researchers includes this theme.

The strategies adopted by the embedded researchers include: (1) identifying challenges in the host organisations, (2) utilising research experience, (3) implementing research evidence, (4) disseminating findings, identifying future research areas, and applying for funding, (5) presenting and publishing findings.

### Identifying challenges in the host organisations

Participants agreed that the research skills of the embedded researchers are essential to the process of identifying the practical challenges facing the research sites. For instance, an embedded researcher used their research skill to unravel the root cause of the challenges facing a school (site three) through a thorough investigation by developing and conducting relevant research with the students and the teachers.

*“[…] the GSCE reforms of the time that was taking place, it was causing a significant amount of stress and pressure for the teachers. In the first instance, teachers were having to grasp new skills at work, they were having to understand the new curriculum and subject knowledge. Some of the teachers weren’t particularly strong, there was a level of undue pressure and stress being put on the students, so pupils nationally were having to learn lots of different contents, they were sort of taken away the security blankets of things like modular testing in course work and what that meant was that students will now have to recall so much more knowledge in exam conditions”****[TR1site3]***.

Following the identification of these challenges, research-based recommendations were offered through the co-production research. By using research evidence to help tackle the school’s challenges, the researcher bridged the gap between the discovery and implementation of research-based evidence.

### Utilising research experience

It is worth noting that the embedded researchers used their research experience to inform their host organisations of relevant existing and newly co-produced research evidence. The embedded researchers’ research-related expertise and the time they spent searching for relevant evidence were both seen as useful to the public health practitioners and other stakeholders.

*“The beauty is that because it is their bread and butter, doing reviews and searching for evidence […] one of the things the embedded researcher did to help me with it was to do that literature review [..] it would have taken me much longer [..], so that’s the benefit [..] it is their strength and their experience and skills which they have got and which we may not have and the time to do it which we may not also have because we are constantly under the treadmill” [****PHP1site1]***.

It was evident that the practitioners’ busy work schedules often restrict their ability to develop and implement their own research skills. Thankfully, the embedded researchers were able to assist the practitioners by using their research skills to overcome research-related challenges, and in the process taught them how to look for research evidence effectively. This, therefore, facilitates the implementation of evidence-based practice. The implication of this is that practitioners’ lack of research skills and time would have served as a barrier for evidence-based practice in the research sites.

It was clear that the research-based evidence searched for, or co-produced by the embedded researchers and the public health practitioners including other stakeholders was used to inform practice and make positive changes. Evidence showed that the embedded researchers had informed the host organisations of relevant research evidence and had used their research experience and skills to make research-based recommendations. In other words, the embedded researchers made valuable research evidence, and knowledge accessible. As such, this brought about desirable changes that improved service and delivery in the research sites.

***“****So the way this works here is that you do the final report which has the recommendations in form of what we feel there should be changes to in practice, and that goes to their public management team and then they will look at that”****[ERsite2]***.

Furthermore, the embedded researchers also discussed how they helped make positive on-site changes occur. For instance, an embedded researcher from site two reported that positive changes were made in practice after developing recommendations in the form of a report submitted for management’s approval. It was clear that the practitioners take evidence-based advice from the embedded researcher to improve the quality of the services being offered to the public. Thus, this closes the gap between research evidence and its implementation.

### Implementing research evidence

The interviews inquired as to how research-based evidence was translated into practice at the four research sites. As the interview process continued, it became clear that desired changes and improvements were achieved through the on-site application of research-based evidence. The results showed that across the four research sites, this process did indeed happen.

*“[..] as it is very much about kind of being a resource to implement the recommendations and embed kind of the key findings from the research, again my role is trying to get some of these things into practice really so its embedded research but the main one of the main things is around embedding the recommendations as well, so that’s sort of work my role is around doing” [****ERsite1]***.

***“*** *[..] at the same time, it also helps the researcher coming in to understand what goes on in practice so that you don’t just go and conduct a piece of research that goes on the shelves. [..] So we would then need to weigh the evidence and the circumstances under which we are going to implement an intervention but we still take advice from the researcher on the evidence of what works. They could advice on what works [..] It’s more about the outcome of research being used to influence practice for quality improvement” [* ***PHP1site1]***

*“There are changes that are made with how they recruit their staff for the delivery staff […] that changes were made and that was in practice, and they also kind of put it in a set of recommendations as to the ones to be delivered in schools”****[ERsite4]***.

Participants reported that the embedded researchers recommended existing research evidence, co-produced research evidence with the intent of informing practice, and also used relevant evidence to help improve service and delivery. In other words, the role of embedded researchers provided accessibility to research-based evidence that was utilised to develop solutions to on-site challenges and create positive change.

### Disseminating findings, identifying future research areas, and applying for funding

The embedded researchers reported that having to present reports to diverse audiences prompted them to produce easily understandable, user-friendly reports that did not rely heavily on academic language.

*“[..] so I have quarterly reports that I have to produce which has to be user-friendly and appeal to a various range of agencies within the organisation [..] we had, basically we have had quite a few different presentations to different kind of groups or the senior management team and departmental teams and things which was about and sharing the results and recommendations, we have follow-ups sort of things from that” [****ERsite1]***.

*“[..] Yeah, just into writing report so she will do like verbal update or she provides like some blueprints in an email****” [PHP5site2]***.

The reports created by the embedded researchers avoided scientific terms that might be difficult for public health practitioners and other stakeholders to understand. Furthermore, practitioners and other stakeholders were informed of relevant research evidence in an unambiguous way. It is important to add that it would have been difficult for the embedded researchers to appropriately simplify their language if they had not had the opportunity to spend time on-site becoming familiar with the language used by the practitioners and stakeholders.

The participants also reported that the embedded research projects effectively discovered potential areas for future research. By making suggestions regarding future research, the embedded researchers furthered each host organisation’s potential to engage in relevant, change-creating research.

*“[..] then the research outcomes were used to inform the next phase, so obviously that was the first phase, which we felt was really successful and worked really well, so then we took those sort of the things we learnt to the next phase”****[ERsite3]***.

For example, an embedded researcher from site three (school) stated that the first phase of their embedded research project was such a success that the findings of the first phase informed the direction of the second phase, thereby ensuring continuous research activities in the school.

Furthermore, participants agreed that the outcomes of the embedded research projects assisted with the application for future funding.

*“[..] the results of the work that we did has been kind of used in terms of future funding opportunities, for providing data, providing kind of context information that was used in sort of proposals and in bids pushing and for applying for future funding”****[ERsite1]***.

It was evident that the presence of the embedded researchers in their host organisations encouraged the push to apply for funding to develop projects. This, therefore, facilitates continuous engagement in research activities. The practitioners felt that the role of the embedded researchers is crucial to producing funding applications and program development.

### Presenting and publishing findings

Once embedded researchers succeeded at co-producing relevant on-site research evidence with practitioners and other stakeholders, and offering practical solutions to on-site challenges, it became clear that it would be necessary to present and publish the outcomes of the projects. Consequently, embedded researchers used their academic skills to publish the findings with practitioners and other stakeholders as co-authors. One of the benefits of publication is that published research can inform the host organisation, and other organisations facing similar challenges. Another significance of the role of embedded research pertaining to this, is that as the embedded research project is co-produced by both the embedded researcher and the host organisation, the findings from the research are jointly owned by both parties. This also assisted in integrating research into the host organisations culture.

*“We wrote a book chapter with their names on the published book chapter. We got all of them involved with the writing of the chapter [..] that makes a sort of massive difference*” ***[ERsite3]***.

*“We co-authored a chapter of a book. We used the findings to create a book chapter but all of us has input into it including the researchers”****[ST2site3]***.

For example, participants from site three (school) reported that a book chapter based on co-produced research that they had worked on with the embedded researcher had been published [43]. Co-produced and co-published research evidence informs the school and research community of the institutional value of embedded research projects. The embedded researcher from site three (school) added that the names of the students and staff involved in the research and writing processes were included in the book chapter. The book chapter was co-edited by both an academic and a public health consultant. This publication has made a tremendous positive difference to how a school labelled as ‘deprived’ views itself. Indeed, being involved in the co-production of valuable research has encouraged both students and teachers.

To further explore how embedded researchers can inform public health practice, the participants were asked whether any other evidence-sharing processes had been used by the embedded researchers. The embedded researchers in this study were connected to more than one organisation. Consequently, they have access to organisations with information that can benefit public health practitioners and other stakeholders. The participants felt that participating in other organisations helped the embedded researchers fulfil their role as the discoverers and sharers of information. The participants viewed this role of the embedded researcher in their sites important as it informs them of the latest research evidence and activities in the field. This could also be seen as a way to sustain evidence-based practice in the sites. As the practitioners are regularly informed of the latest relevant evidence by attending research-based programmes, it facilitates the integration of research into the host organisations’ culture.

*“When I see opportunities for conferences or local events, I will send an email or circulating them, there might be public health conference, it might be a Fuse conference that’s linked in erm linked in heavily with the thing we have worked on and I circulate that to the staff member, to say here is an opportunity”****[ERsite2]***.

For instance, an embedded researcher from site two stated that she regularly informed the practitioners of programmes and events presenting research relevant to their practice. By attending such events, practitioners can stay informed and up to date and are more likely to make changes to their practice based on timely research evidence. Consequently, the findings of this study indicate that staying familiar with the latest relevant research is one of the ways to close the gap between the discovery and implementation of research-based evidence.

Overall, it was evident that the embedded researchers’ ability to inform the organisations with relevant co-produced research evidence, and the ability to identify relevant information and opportunities and then circulate these to public health practitioners and stakeholders helped to inform the sites in creating relevant, research-based changes to benefit their public health practices. The positive outcomes they generated indicate that the role of embedded researchers can seriously contribute to closing the gap between the discovery and implementation of research-based evidence in the research sites.

### Critical reflection

Twelve out of seventeen participants across the four sites discussed this theme as part of the role of the embedded researcher in their respective organisations. Participants felt that critical reflection was an important process an embedded researcher must engage in throughout the ‘journey’ of becoming an agent of closing the gap between research evidence and its implementation in practice. The identified strategy adopted by the embedded researchers within this theme is continuous reflection.

*“I constantly reflect on my role to know what I am doing right, and what can be done differently”****[ERsite1]***.

*“I have to spend really more time reflecting”****[ERsite2]***.

*“It might be while you drive home [..] might be in the shower [..] might be when I take the dog out for a walk and tea time to reflect because you do need time to reflect on your research, on your methodology [..] about what the findings need to show [..] at times my bag is full of paper everywhere, millions of notes in here and I have to open and jot down some questions so that I won’t forget them because they are so important”****[ERsite4]***.

*“I think it’s always good to sort of like reflect on what we have done, how we do things I personally want to think about whether I could have done things better […] so I think it’s quite important to sort of reflect on how you have done things, and how you could do things in the future, like what lessons you have learnt, I think it’s important to sort of reflect, to sort of think more about how you have done things and whether it could be practiced in the future”****[ERsite3]***.

Overall, the participants agreed that reflection helps embedded researchers assess their roles and constantly improve their work. Therefore, reflection is crucial to successfully co-producing research and closing the research implementation gap.

## Discussion

All participants, irrespective of their age, working experience and education, acknowledged that the relationships between the people involved in an embedded research project are crucial to the project’s success. This is in keeping with those made in previous studies that have concluded that building and maintaining mutually beneficial relationships with practitioners and other stakeholders significantly helps embedded researchers co-produce public health knowledge in non-clinical settings [33, 44]. The study participants were also unanimous in their view that the ‘embeddedness’ of the researchers, or the degree to which they become part of or spend time within the host organisation, is significant. A higher degree of embeddedness appears to lead to the development of beneficial relationships and also helps researchers develop a better understanding of organisational contexts, that in turn leads to the development of effective solutions and useful, co-produced research. Notably, becoming embedded to a significant degree helps others see the researchers as part of the team. Previous studies have also indicated it is the duty of the embedded researcher to become part of the host organisation by working collaboratively with practitioners and other stakeholders [17, 45].

Although the amount of time each embedded researcher spent within their host organisation varied, the interview data gathered from all sites confirmed that embedded researchers felt they were able to develop meaningful relationships with the host organisation. The National Institute for Health Research (NIHR) embedded research team reported similar findings and observed that the amount of time spent within an organisation can depend on the intensity of a project [46].

Among other strategies, informal conversations with the practitioners and other stakeholders also assisted the embedded researchers to build relationships. This was confirmed only by the embedded researchers in case study sites two and four who had worked in the host organisations for more than three years. This might be because the embedded researchers from the local authority (site two) and the sports organisation (site four) had worked and familiarised themselves with the members of the host organisation staff. Consequently, this could have facilitated easier informal conversations, unlike the embedded researcher in site one who has just spent seven months in the site. This confirms that it takes time for embedded researchers to build trustworthy relationships in the host organisation and they recommend an ‘introductory period’ of a minimum of three months for familiarisation before an embedded research project starts [39]. This was beneficial to the three case studies explored in an earlier study as it allowed the embedded researchers to familiarise themselves with their host organisations and as well build relationships with the host organisations’ staff [39]. This also aligns with the view of other scholars that an ‘introductory period’ is important before the commencement of an embedded research project [44]. It is worth noting that the practicability of an ‘introductory period’ may depend on the agreement between the parties involved.

Furthermore, embedded researchers must build relationships not only with practitioners and other stakeholders, but also with their academic supervisors. Having a successful relationship with the academic supervisor can help the embedded researcher overcome the challenges that arise as a consequence of having a dual affiliation and needing to manage diverse expectations and competing interests. The embedded researchers interviewed in this study had the support of their academic supervisors. Thanks to the vast experience of their supervisors, they are often excellent at mitigating unforeseen challenges. Indeed, among other factors, the success of an embedded researcher depends on the relationship between the researcher and his or her academic supervisor [13, 39].

The interview participants recounted that it is important to work together to co-produce relevant research which is useful to the organisations. Other scholars have similarly concluded that embedded researchers work with members of their host organisations to identify, plan, and conduct research that will meet the needs of the organisation [36]. By working collaboratively, embedded researchers were able to train the practitioners and other stakeholders and improve their ability to help co-produce meaningful and valuable research that can be used to implement evidence-based adjustments to on-site practices.

The findings of this study indicate that working together produces meaningful research and also teaches practitioners and other stakeholders who assist embedded researchers, how to conduct research. Similarly, an earlier study concluded that embedded researchers encourage practitioners and other stakeholders to participate in research activities and increase an organisation’s capacity to conduct research [17]. In other words, the collaborative work that accompanies embedded research helps close the research implementation gap. However, it was noted in this current qualitative inquiry that having the right researchers assisted in carrying out the projects successfully. This is similar to an earlier study that argue that having the right combination of researchers and practitioners in co-production is crucial to the success of such project [13]. Also, other scholars pointed out that not all researchers have the relevant skills to conduct co-produced research [17]. Therefore, it is essential to have the right combination of researchers, practitioners, and other stakeholders while working together to co-produce research to ensure its success.

Based on the current qualitative inquiry, the role of the embedded researchers includes informing practice by making recommendations and positive changes that utilise both existing and newly co-produced research evidence. Doing so makes research evidence more accessible to public health practitioners and other stakeholders and ultimately improves service and delivery. An earlier study similarly revealed that informing practice has been identified as a way by which embedded researchers communicate new and existing relevant research evidence and integrate research findings into practice [3].

As discussed earlier, two of the factors responsible for the gap between the discovery and implementation of research evidence are the disparity between the language spoken by the researchers and practitioners and the complexity of the language spoken by researchers, which is often include scientific jargon. Such complex language can be difficult for practitioners to understand or lead to ambiguities in interpretation [12]. To discover whether language differences was an issue in this study, the interviews included questions regarding how research evidence and recommendations were communicated to public health practitioners and other stakeholders. These questions were designed to create an understanding of how the embedded researchers had communicated. The interviews revealed that the embedded researchers communicated research outcomes and recommendations effectively to the practitioners by using simple, unambiguous language. Using such language helped make research evidence more accessible to the practitioners.

Providing evidence for reports and future funding applications was identified as an important part of the embedded researchers’ work within their host organisations [17, 47]. The interview participants agreed that the researchers sometimes helped secure funds needed to conduct research at the host organisation. Doing so encouraged each host organisation’s staff to participate in research that could prove useful to the organisation in the future.

Critical reflection helps embedded researchers evaluate the role they play within their host organisation and keep track of their progress [33, 48]. In other words, reflection helps researchers identify and improve upon the areas that are not meeting expectations and discover what approaches are working successfully. This corresponds with the findings from this current qualitative inquiry. The interview participants acknowledged that the embedded researchers continuously reflect on their role and their work in order to identify what is and is not working. This assists embedded researchers to think of ways to apply acquired learning to daily on-site practice to improve their role in the co-production of research to bridge the gap between research evidence and its implementation in public health practice.

### Limitations of the study

One of the limitations of this study was the sample size. A total of 17 participants was recruited for this study, although the sample size would have been larger than 17 but for the COVID-19 pandemic. Another consideration of this piece of work, being qualitative research, was subjectivity. The information provided by the participants was based on their point of view. Hence, it might be difficult to objectively verify the qualitative information provided to ensure that accurate information was provided by the participant regarding the phenomenon of interest. Nevertheless, some practical measures were undertaken to ensure the credibility of this work. Data triangulation and site triangulation [49] were adopted in this study. These were done to increase the confidence in the outcome of the qualitative multi-site case study.

## Conclusion

Overall, the success that the embedded researchers experienced, including building relationships, co-producing research, translating research into practical changes, evaluating projects, and informing future public health practices as well as future research, justifies increasing the amount of embedded research being conducted in public health practice. Embedded researchers also bring the tremendous benefit of strengthening the research capacities of public health practitioners and other stakeholders by providing research-based training and support. Such developments have the ability to prove the potential of embedded research projects. Finally, the relevant research-based recommendations made from the co-produced research guided by the embedded researchers are used to inform practice. The positive outcomes generated by the embedded research process indicate that embedded researchers can meaningfully contribute to closing the gap between the discovery and implementation of research evidence.

## Data Availability

The datasets generated and/or analysed during the current study are not publicly available. They are available from the corresponding author on reasonable request, subject to approval from the Teesside University School of Health and Life Sciences Research Governance and Ethics Committee.

## Appendix

### Interview schedule for embedded researchers

#### Role identification and background information about the embedded research initiative

1. What is your role in your organisation? ***Prompt****- *Job title, Daily task, Responsibilities. B) How long have you been in this role? C) Can you tell me about your background and what you do? ***Prompt*** -The journey so far- How do you get to where you are now?  D) As an embedded researcher where is your academic affiliation?
2. How long has your embedded research initiative been going on in your organisation? B) Do you know the rationale for employing an embedded researcher in your organisation? C) Who funds your project? D)What is the management arrangement?

#### Moving on to look at the embedded research initiative more specifically

3. What is the aim of the embedded research project you are involved in? B) How many hours/days do you spend in your host organisation in a week, and in the academic institution?  C) Why? D) How often do you contact your academic supervisor?
4. How has embedded research gone so far in your organisation?  B) How many people are involved in the co-production/embedded research you are involved in? or who do you work with? C) How many embedded researchers are involved in the project?***Prompt**** - *How many professionals/stakeholders?
5. What are your views and experience of embedded research?***Prompt***- what have you learnt? What, if anything, has helped?  (Why do you say that?) What, if anything, has been more difficult or challenging? (Why do you say that)? What difference has embedded research made in your organisation?  (so if embedded research has been useful, why and how?)

#### Looking more specifically at the role of the embedded researcher in the organisation

6. What is your role, as an embedded researcher in bridging the gap between research evidence and its implementation in practice? ***Prompts -*** How do you inform practice with research evidence?  How do you communicate research evidence to practitioners and other stakeholders to facilitate its use in practice? B) Does your role involve the translation of research evidence into practice? If yes, what is the process? can you please cite an example? What evidence-sharing methods or processes do you use?
7. Can you think of any changes in practice/policy as a result of research evidence being used? ***Prompt*** – What role did you play? Who was involved? What changed? How? For who?
8. Tell me what you think are the benefits of working as an embedded researcher? Why do you say that? B) How do you manage the dual affiliation? ***Prompt***-what are the benefits (What has helped?) and also what are the challenges?
9. Tell me what you think are the challenges of working as an embedded researcher? ***Prompt ***- Why do you say that? B) What are the barriers to data sharing, if any?
10. Do you think building mutually beneficial relationships with the host organisation staff is important to the success of an embedded research project? If yes, Why? B) How do you build relationships with the host organisation’s staff?
11. Can you cite an example of where you have built practitioners and other stakeholders’ confidence in conducting their own research?
12. Does your role requires managing research funds? If yes, how do you manage this?
13.  How often do you reflect on your role? ***Prompt-*** To know what works and what needs to be improved?  Why is this important?
14. Do you think the development of a toolkit on the role of embedded research in bridging the gap between research evidence and its implementation in public health practice would be useful? If yes, Why and how do you think it could be used in practice?”
15. Any top tips for other researchers considering embedded research?
16. Please don’t mention names, but can you think of any potential participants- people you are working with or have worked with that you can pass on the details of this research?  B) Would you be happy to be contacted afterward to circulate details of this research to those you have identified, to see if they will be willing to participate in this research?
